# Cytotoxicity Activity and Druggability Studies of Sigmasterol Isolated from Marine Sponge *Dysidea avara* Against Oral Epithelial Cancer Cell (KB/C152) and T-Lymphocytic Leukemia Cell Line (Jurkat/ E6-1)

**DOI:** 10.31557/APJCP.2020.21.4.997

**Published:** 2020-04

**Authors:** Melika Nazemi, Mostafa Khaledi, Mahdi Golshan, Masoud Ghorbani, Mohammad Reza Amiran, Alireza Darvishi, Omid Rahmanian

**Affiliations:** 1 *Persian Gulf and Oman Sea Ecological Center, Iranian Fisheries Research Institute, Agricultural Research, Education and Extension Organization (AREEO), *; 6 *Department of Food and Drug Administration, Hormozgan University of Medical Sciences, Bandar Abbas, *; 2 *Marine Pharmaceutical Science Research Center, School of Pharmacy, Ahvaz, Jundishapur University of Medical sciences, Ahvaz, *; 3 *Iranian Fisheries Research Institute, Agricultural Research, Education and Extension Organization (AREEO), *; 4 *Pasteur Institute of Iran, *; 5 *Department of Hepatitis and AIDS, Pasteur Institute of Iran Tehran, Iran. *

**Keywords:** Marine sponge, cytotoxicity, KB/C152, Jurkat/ E6-1, PASS, molecular docking, ADMET, GC-MS

## Abstract

**Background:**

Marine sponge is a rich natural resource of many pharmacological compounds and various bioactive anticancer agents are derived from marine organisms like sponges.

**Methods:**

studying the anticancer activity and Drug ability of marine sponge *Dysidea avara *using Cell lines oral epithelial cancer cell (KB/C152) and T-lymphocytic leukemia cell line (Jurkat/ E6-1). Marine sponge was collected from Persian Gulf. Several analytical techniques have been used to obtain and recognize stigmasterol, including column chromatography, thin layer chromatography, and gas chromatography-mass spectrometry. The PASS Prediction Activity was used to investigate the apoptosis-inducing effect of stigmasterol. The cytotoxic activity of stigmasterol was examined using yellow tetrazolium salt XTT (sodium 2, 3,-bis (2methoxy-4-nitro-5-sulfophenyl)-5-[(phenylamino) carbonyl]-2H-tetrazolium) assay. The stigmasterol were docked within the protein tyrosine kinase (PTKs) (PDB code: 1t46) and epidermal growth factor receptor (EGFRK) (PDB code: 1M17). Also, the pharmacological characteristics of stigmasterol were predicted using PerADME, SwissADME, and Molinspi ration tools. Apoptosis-inducing effect of stigmasterol indicate the stigmasterol in terms of the possibility of apoptosis in cells.

**Results:**

The apoptosis inducement results of known stigmasterol were determined by PASS on-line prediction. The compound exhibit potent cytotoxic properties against KB/C152 cell compared to Jurkat/ E6-1 cell. The stigmasterol showed the cytotoxicity effects on KB/C152 and HUT78 with IC50 ranges of 81.18 and 103.03 μg/ml, respectively. Molecular docking showed that, stigmasterol bound stably to the active sites of the protein tyrosine kinase (PTKs) (PDB code: 1t46) and epidermal growth factor receptor (EGFRK) (PDB code: 1M17).

**Conclusion:**

The compound showed desirable pharmacokinetic properties (ADME). This provided direct evidence of how a prospective anti-cancer agent can be stigmasterol. The preclinical studies paved the way for a potential new compound of anti-cancer.

## Introduction

Marine sponges are one of the significant invertebrate groups living on the marine ecosystems benthic. Although there is no effective physical defense and attached to the bottom by marine sponges. The way to prevent predators is to produce bio-active compounds. It has been noted that many biologically active compounds from sponges inhibit cell growth, so they are exciting natural products to obtain new cancer drugs (Sipkema et al., 2005).

Today, many anticancer drugs have been extracted or derived from bioactive compounds, including Antiproliferative drugs such as doxorubicin, Bleomycin and etc., which are used in the chemotherapy (Jimeno et al., 2004). Several bio-natural compounds were included in clinical studies and clinical trials for evaluation with anti-proliferative activities obtained from marine sponges (Becerro et al., 2003; de Caralt et al., 2010). *Dysidea avara* is purple, massive shape, and heavily convoluted sponge, frequently observed at depths between 1 and 100 m (Hooper and Van Soest, 2002). In the present studies, we used thin-layer chromatography, column chromatography, and gas chromatography-mass spectrometry to acquire and recognize stigmasterol in the extract of acetone. Cell lines of the oral epithelial cancer cell (KB / C152), T-lymphocytic leukemia cell line (Jurkat / E6-1) screened the cytotoxicity operations of extracted stigmasterol.

## Materials and Methods

*Collection of marine sponge*

The sponge was manually gathered in the coastal waters of the Hengam Island Persian Gulf using SCUBA at depths of 15-30 m: N 26 ° 36’43’’ and N 26 ° 41’15’’, E 55 ° 54’40’’ and E 55 ° 54’55’’.’ The species was identified by a taxonomist at the Persian Gulf and Oman Sea Ecological Research Institute, Bandar Abbas, Iran.

*Extraction and isolation*

The marine sponge collected was transported in an icebox to the laboratory and was washed twice with fresh water and distilled water. The sample was cut into tine pieces (1 cm3) and dried using freeze drier. After drying, powdering and holding until extraction at - 24°C. Approximately 200 g of powder was obtained at room temperature with acetone (600 cc) for 72 h (Nazemi et al., 2014). The acetone solution was filtered in the rotary vacuum evaporator (Whatman No 1) and dried at 39^o^C. The crude acetone extract (27.12 g) was separated into 128 fractions, C1–C128, by chromatography (Silica gel 0.2-0.6 mm mesh, 500 g, 2 cm × 70 cm) using gradient mobile phase of N-hexane-ethyl acetate (100:0–0:100, v/v) and ethyl acetate -methanol (100:0–0:100, v/v). 

The fraction C36 (Yield 90 mg) that received positive response for Terpenoids by Vanillin-sulphuric acid reagent was selected and was then separated into eight sub fractions, C36A–C36j, by Silica gel (0.2-0.6 mm mesh, 500 g, 0.5 cm × 50 cm) using eluent of N-hexane-ethyl acetate (100:0–0:100, v/v) and ethyl acetate -methanol (100:0–0:100, v/v). 

Then, the sub fraction C36E (20 mg) that had a positive response for Terpenoids subjecting it on TLC eluting using Methanol: chloroform: N-hexane (10:70:20 v/v) after eluent the spot that received positive response for Terpenoids by Vanillin-sulphuric acid reagent, was selected for determining the use of GC–MS. 

The Gas chromatography-mass spectrometry (GC-MS) spectrum was taken on GC-MS (Agilent7000 Series Triple Quad GC/MS MainFrame؛). The column part number 19091s-436 (60 m × 0.25 mm, film thickness 0.25 μm) was used for GC-MS. Also, helium gas was used as a carrier gas at a flow rate of 1.0 mL / min. The electron ionization mode with ionization energy of 70 eV was used for MS detection, with a mass range of 50–650 m / z. The compound was recognized using standard information in the Wiley 7.0 library by its retention time and mass fragmentation pattern.

*The probability of apoptosis inducing effect*

Apoptosis-inducing impact of stigmasterol has been studied using Prediction Activity Spectra of Substances (PASS). Accordingly, PASS is a free online tool (http://www.pharmaexpert.ru/passonline/predict.php) and for assessing the overall biological potential of an organic drug-like molecule, it offers concurrent predictions of various kinds of biological activity based on the target compounds ‘ chemical properties (Lagunin, 2010). Prior to in vitro and in vivo testing, PASS can be used to monitor the biological activity profiles for virtual molecules.

*Cytotoxic properties*

The oral epithelial cancer cell (KB/C152), T-lymphocytic leukemia cell line (Jurkat/ E6-1), and human embryonic kidney (Hek293) cells were bought from Pasteur Institute of Iran, Tehran, Iran, and the tests were performed in the laboratory of department of hepatitis, HIV, and Bloodborne Viruses. The Cytotoxic effects of stigmasterol was evaluated using KB/C152, Jurkat/ E6-1, and Hek293 cells. The KB/C152, Jurkat/ E6-1, and Hek293 cells were maintained in RPMI (sia) with 10% Fetal Bovine Serum and antibiotics (about 100 Unit/mL of penicillin, 100 mg/mL of streptomycin). Also, 25,000 Cells were seeded in 96-well plates containing medium with 2, 10, 50, 100, and 200µg/ml of stigmasterol. The plates were kept for 24h at 37 °C in 100% humidity with 5% CO_2_ and 95% O_2_.

After 24h, 100 µL of XTT was added to each well, and for an additional 4h, plates were incubated at 37°C. The mean of triplicate experiments for each dosage was used to score the median inhibitory concentration (IC_50_) and the combination index. Concentration of the reduced XTT was quantified by measuring the absorbance at 490 and 690 nm in an ELISA reader to estimate the IC_50_ values. 

All tests were conducted in triplicate, and cytotoxicity was expressed as IC_50_, which was calculated using GraphPad Prism 5 software, and submitted six independent experiments with three replicates for each concentration as mean ± SEM. 

*Molecular docking studies*

In the silico automated docking research, an AutoDock Tools (ADT) version 1.5.6 and version 4.2.5.1 docking program were used in house batch script (DOCKFACE) (Morris et al., 2009).

AutoDock Tools was used to remove crystal water, by adding the polar atoms of hydrogen and Gasteiger charges to each atom, and merging the non-polar atoms of hydrogen. Docking studies were performed by Intel^®^core i5 CPU (2.53 GHz) with Windows-7 operating system. 

The docking study of Stigmasterol was performed, which were docked within the protein tyrosine kinase (PTKs) (PDB code: 1t46) (Mol et al., 2004), and epidermal growth factor receptor (EGFRK) (PDB code: 1M17) was obtained from the Protein Data Bank https://www.rcsb.org (Stamos et al. 2002).

The active protein region of the targets has been selected based on previous studies (Ali, 2007). The binding site on 1t46 was defined by a grid system of 82 × 80 × 80 Å3 and 86 × 82 × 80 Å3 for 1m17 with a grid Spacing of 0.375 Å that was originated at the center of grid box. Docking simulations with a rigid receptor structure were allowed to be flexible in the ligand structure using the Lamarck Genetic Algorithm (LGA).

The Lamarckian genetic algorithm has been implemented with the following protocol: trials of 150 runs, energy evaluations of 50,000,000, a limit of 30,000 generations, a population size of 200, a mutation rate of 0.02, a crossover rate of 0.8, and an elitism value of 1. The docking outcomes were assessed by sorting out the docking energy predicted by docking conformations. The protein ligand interaction was assessed using Chimera 1.13 software and also online web-based Plip software.

At last, the resulting docked ligand conformation was evaluated in terms of energy, hydrogen bonding, and hydrophobic interaction between stigmasterol and receptors, to determine the binding mode of the effective inhibitors.

*Pharmacological Properties of ADMET*

The pharmacological characteristics of stigmasterol were predicted using PerADME, SwissADME, and Molinspiration tools (Istiqomah et al., 2019).

*Statistical analysis*

All the data were evaluated using one-way ANOVA, followed by various comparative testing by Tukey – Kramer’s. P values < 0.05 were considered as statistically significant for the stigmasterol anticancer operations.

## Results

*Isolation and verification of stigmasterol*

In column chromatography, the acetone extract was packed and eluted with the distinct solvent system. Based on the TLC profile, more than 128 fractions have been gathered from column chromatography. The Fraction C36 (Yield 90 mg) that received positive response for Terpenoids by Vanillin-sulphuric acid reagent was selected and then separated into ten sub fractions, C36A–C36j, by Silica gel. The fractions of C36F were subjected to TLC for isolation of active principle. After TLC run, the band that received positive response for Terpenoids producing pink to purple color with Aniline test, wad punched, dissolved in acetone, and finally filtered, and we obtained yellow crystals of compound (4.5 mg) and then Comparing GC-MS with Wiley 7.0 library (Figure 2) verified the compound’s identity. 

*Apoptosis-inducing effect of stigmasterol*

The probability of the apoptosis-inducing effect of stigmasterol from *Dysidea avara *was shown in Table 1. The activity of the apoptosis agonist was discovered to be 0.752. The results indicate the stigmasterol in terms of the possibility of apoptosis in cells. 

*Cytotoxic properties of the isolated compound*

The isolated compound was subjected to 24 h incubation in different concentrations (0, 10, 50, 100, and 200 μg/mL) on cell viability of KB/C152, Jurkat/ E6-1, and Hek293 cell lines that are presented in Figure 2. Subsequently, XTT assay examined the capacity of cell survival. Cell survival rate after the treatment with 200 μg/mL stigmasterol was significantly decreased to 15.67 percent. Stigmasterol showed the high dose-dependent inhibition of the cell population with a half-maximum inhibitory (IC_50_) value of 69.59 μg/mL. The determined half-maximal activity concentration (IC_50_) of the stigmasterol on the Jurkat/ E6-1 was 86.44 μg/mL for 24 h. The cell survival rate after treatment with 200 μg/mL stigmasterol significantly decreased to 25.95 percent. KB / C152 and Jurkat/ E6-1 cell viabilities considerably decreased following the treatment with 20, 50, 100, and 200 μg/mL stigmasterol, as shown in Figure 3.A and B, compared to negative control (0 μg/mL stigmasterol).

The IC_50_ values on noncancerous Hek293 cell line were about 202.61 μg/mL for 24. Hek293 cell viability considerably increased following the treatment with 2, 10, and 20 μg/mL stigmasterol, Compared to negative control (0 μg/mL stigmasterol) (Figure 3C). Overall, the cytotoxicity assay depicted that, the stigmasterol inhibited the proliferation of the cell lines oral epithelial cancer cell (KB/C152) and T-lymphocytic leukemia cell line (Jurkat/ E6-1) in a dose-dependent manner, whereas it had minimal cytotoxic effect on the human embryonic kidney cells (Hek293).

*Molecular docking properties*

The stigmasterol showed binding energy value of -9.98 kcal/mol with the docked EGFRK (1M17). The stigmasterol can interact with two active site amino acids namely LEU- 694, LEU- 694, VAL-702, LYS-721, THR-766, LEU-768, LYS-242, MET-769, LEU-820, LEU-820, GLU-738, and GLU 738 with 2 hydrogen bonds. In stigmasterol, C6H8-O-H interact with the O-CO GLU-738 and can form a hydrogen bond with the bond lengths of 2.13 Å and 1.86 Å. The binding interaction of the stigmasterol with the EGFRK is shown in Figure 4 and Table 2.

The stigmasterol exhibit binding energy value of -11.97 kcal/mol with the docked PTKs (1T46). The Stigmasterol can interact with 12 active site amino acids namely ILE-571, PRO-573, GLU-640, VAL-643, LEU-644, LEU-644, ILE-644, ILE-653, VAL-654, ILE-808, ASP-810, and ILE-571 with one hydrogen bond. In stigmasterol, C6H8-O-H interact with the C=O of ILE-571 and can form a hydrogen bond with the bond length of 2.15 Å. The binding interaction of the compound with the 1M17 and 1T46 is shown in Figure 5 and Table 1.

*ADME analysis*

The stigmasterol was analyzed for its pharmacological properties and toxicity using various software. The details of stigmasterol physicochemical properties are shown in Figure 6 and Table 3, 4, and 5.

In Figure 6, the pink color area represents the optimal range for -0.7 ≤ Lipophilicity ≤ +5.0, 150 ≤ MW ≤ 500 g/mol, 20 ≤ TPSA ≤ 130, log S ≤ 6, saturation ≥ 0.25, and flexibility ≤ 9 rotatable bonds (Daina et al., 2017). In the stigmasterol, the compound is not predicted as orally bioavailable, because it is poorly soluble (-7.46) and has a high Lipophilicity (8.56).

The tests included PAINS test, Brenk test, Veber test, and Egan test. PAINS test and Brenk test were conducted to find out if there were ligand parts that could provide a bogus biological response. Stigmasterol carcinogenicity-mutagenicity testing was also accomplished using perADMET website. Table 4 showed the carcinogenicity characteristics of the mutagenicity of the stigmasterol.

Bioactivity test of the stigmasterol was performed using the Molinspiration online software. Accordingly, Molinspiration is used as the best drug applicants to determine the properties and bioactivity of natural product ligand compounds. The parameters presented in the software are the results of comparing the similarity of the ligand results from the experiments of ligands with high bioactivity properties; for example, ligands, modulator channels, kinase inhibitors, protease inhibitors, and experimental database enzyme inhibitors such as G protein-coupled receptors (GPCR) (Verma, 2014). The findings of the stigmasterol test by using internet software from Molinspiration are shown in Table 4. 

## Discussion

The marine natural products have received considerable attention in the past decades. There are a large number of marine products that have been targeted by pharmaceutical industry. Sponges are the most important producer of bioactive compound in terms of variety. In the present study, the stigmasterol, which is a steroid compound, was extracted from *Dysidea avara *using nonpolar solvents such as petroleum ether and N-hexane (Chaudhary et al., 2011). The stigmasterol has been identified from Navicula incerta (Kim, 2014), the Dragmacidon coccinea marine sponge from the Red Sea (Abou-Hussein et al., 2014), stigmasta-5,24 [28]-dien-3-ol, 5,24 [28]-Stigmastadien-3β-ol, 3β-hydroxy-5,24 [28]-stigmastadiene, and stigmasta-3,5-dien-7-one from the Stigmasterols family of the Condrosia reniformes, Tethya rubra, Tethya ignis, Mycale angulosa, and *Dysidea avara *from the Spanish and Portuguese marine sponges (Paula et al., 2015).

Sponges can release cytotoxic substances to prevent predators and survive through their evolution, as they require water pumping for surviving, and on the other side, they are unable to filter the biofilm or remove barnacles, mosses or corals that cover their surface and leading to their death. Accordingly, they have been able to deal with aggressive organisms that cover their surface and prevent water filtration to compete for survival. Nowadays, thanks to the advancement of the science, such chemical compounds with the potential to destroy living cells, are used as anticancer drugs (Raveendran and Mol, 2009). 

In this study, the cytotoxicity of the stigmasterol has been examined. It has been observed that, the stigmasterol exhibit cytotoxic effects on the oral carcinoma and lymphocytes epithelium cells at the concentrations of 81.18 and 103.03 μg/ml. However, lethal effects have been found on the human embryonic kidney cells. In other word, the fraction containing the stigmasterol can only affect cancer cells. Previous studies conducted on stigmasterol extracted from the Aurora globostellata sponge, showed the inhibitory effect on the growth of breast cancer cells (Sugappriya et al., 2017), and observed that, the stigmasterol and its derivatives extracted from Xestospongia sp marine sponge can result in killing 50% of the lymphocyte cancer cells (K562) at 18/3 to 34/3 μg/ml (Cheng et al., 2016). In another study conducted on the cytotoxic properties of the extract containing stigmasterol (more than 25%) from the Spanish and Portuguese marine sponges on the LLC-MK2 healthy cells (epithelial cells from the kidney of the monkey Macaca mulatta), it has been shown that, Tethya ignis (177.5 μg/ml), Tethya rubra (172.5 μg/ml), Dysidea avar (144.2 μg/ml), and Mycale angulosa sponges (302.71 μg/ml) can exhibit lethal effects (Paula et al., 2015). Accordingly, based on the results of the stigmasterol extraction from the Dysidea avar sponge of the Hengam Island, its fraction showed a lethal effect on the healthy cells at very high concentrations, which is consistent with the results of other studies (Paula et al., 2015; Cheng et al., 2016). Two tests of Weber and Egan were performed to determine the mold oral bioavailability of stigmasterol (Daina et al., 2017). Accordingly, it can be absorbed and distributed with less damage to the body and healthy cells, and can induce its metabolic effect on the cancer cells followed by absorption, distribution, metabolism, elimination, and toxicity (ADMET). Stigmasterol is non-substrates of 5 major isoforms CYP 1A2, CYP2C19 and CYP2D6 but stigmasterol is substrates of CYP2C9 Inhibition of this isoenzyme is certainly one major cause of pharmacokinetics related drug-drug interactions leading to toxic ADME due to accumulation of drug/ metabolites. Therefore, the stigmasterol can be considered as an appropriate anticancer medication candidate to enter the market (Gangopadhyay et al., 2017). The molecular docking of stigmasterol studies exhibit lower binding energy (-9.98 kcal/mol) when docking with EGFRK (1M17) in comparison with the control ligand (Gefitinib). Results of this research indicate that, the stigmasterol may be used as a preventive and therapeutic agent against oral epithelial cancer cells. The findings in silico (docking research, ADMET) and in vitro showed that, the proper drug-like characteristics and potency cytotoxicity of stigmasterol were found.

**Figure 1:**
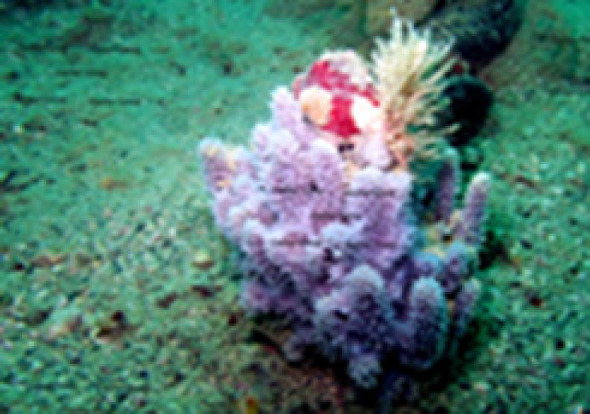
Marine Sponge *Dysidea avara*

**Table 1:** The Results of PASS of the Stigmasterol

Activity	Pa	Pi
Apoptosis agonist	0,752	0,011

**Table 2:** Binding Energy Values of Stigmasterol which were Used for Various Cancer Receptors

Receptors	Compound name	Parameters				
		Binding energy (kcal/mol)	Number of Hydrogen bonds	Amino acid residues	Number of Hydrophobic Interactions	Amino acid residues
1M17	stigmasterol	-9.98	2	GLU-738, GLU-738	9	LEU- 694, LEU- 694, VAL-702, LYS-721, THR-766, LEU-768, LYS-242, MET-769, LEU-820, LEU-820
	Gefitinib	-8.02	3	THR-766, THR-766, MET-679	7	LEU- 694, VAL-702, VAL-702, ALA-719,LEU-768, MET-769, LEU-820
1T46	stigmasterol	-11.97	1	ILE-571	11	ILE-571, PRO-573, GLU-640, VAL-643, LEU-644, LEU-644, ILE-644, ILE-653, VAL-654, ILE-808
	Imatinib	-13.84	4	GLU-640, THR-670, ILE-789, ASP-810	11	VAL-603, ALA 621, LYS-623, LEU-644, LEU-644, ILE-6653, VAL-668, THR-670, TYR-672, LEU-799, ASP-810

**Table 3:** Evaluation Parameters of Lipinski’s rule of Five and Its Extensions from Stigmasterol

Physicochemical Properties		Pharmacokinetics				Drug likeness	
Formula	C29H48O	GI absorption	No	CYP3A4 inhibitor	No	Lipinski	Yes;
Molecular weight	412.69 g/mol						1 violation:
Num. rotatable bonds	5	BBB permeant	-2.74 cm/s	Log Kp (skin permeation)	-2.74 cm/s		MLOGP>4.15
Num. H-bond acceptors	1						
Num. H-bond donors	1	P-gp substrate	No	Medicinal Chemistry		Ghose	No; 3 violations: WLOGP>5.6, MR>130, #atoms>70
Molar Refractivity	132.75	CYP1A2 inhibitor	No	PAINS	0 alert	Veber	Yes
		CYP2C19 inhibitor	No	Brenk	1 alert: isolated_alkene		
TPSA	20.23 Å²	CYP2C9 inhibitor	Yes	Leadlikeness	No; 2 violations: MW>350, XLOGP3>3.5	Egan	No; 1 violation: WLOGP>5.88
Log Po/w (XLOGP3)	8.56	CYP2D6 inhibitor	No	Synthetic accessibility	6.21	Muegge	No; 2 violations: XLOGP3>5, Heteroatoms<2
Log S (ESOL)	-7.46	GI absorption	Low	PAINS	0 alert	Bioavailability Score	0.55

**Figure 2.**
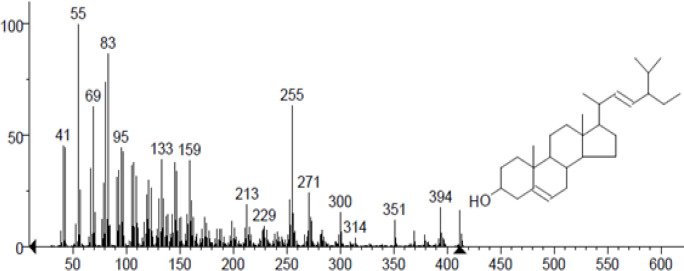
GCMS Analysis of Stigmasterol

**Figure 3:**
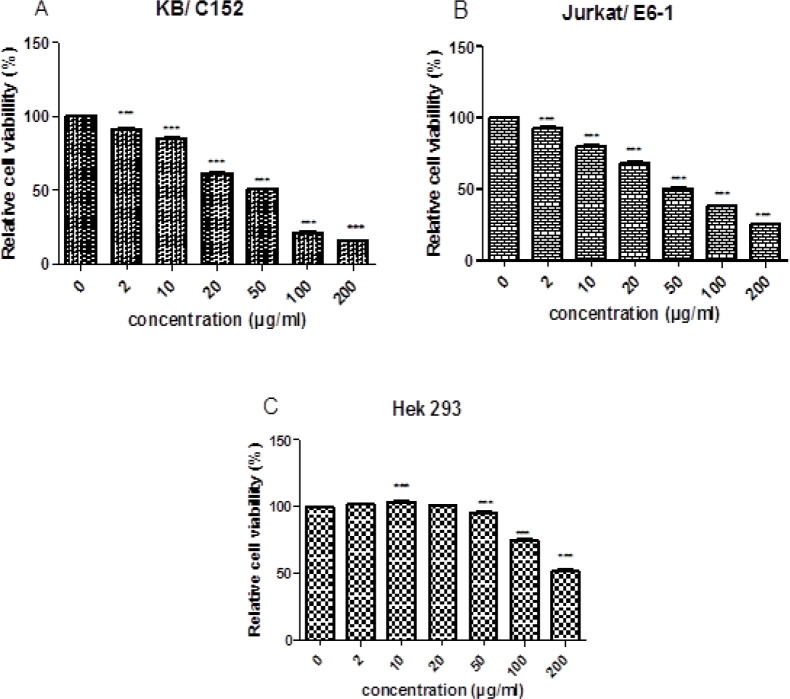
Dose-Dependent Stigmasterol Inhibits (A) KB / C152 (B) Jurkat/ E6-1 (C) Hek 293 Cells Survival. At different concentration (2, 10, 20, 50, 100 and 200 μg/mL), cells were Treated with stigmasterol for 24 h. Bars are meaning ± standard deviation. *P*-values were evaluated with Dunnett's post-hoc test by one-way assessment. ***P<0.001 vs. 0 μg/mL Treatment group of stigmasterol

**Table 4:** Carcinogenicity-Mutagenicity Test Stigmastrol by preADMET

Ames TA 100 (+S9)	negative
Ames TA 100 (-S9)	negative
Ames TA1535 (+S9)	negative
Ames TA1535 (-S9)	negative
Ames test	non-mutagen
Mouse	positive
Rat	positive

**Table 5:** Bioactivity Test by Molinspiration Software

bioactivity	score
GPCR ligand	0.12
Ion channel modulator	-0.08
Kinase inhibitor	-0.48
Nuclear receptor ligand	0.74
Protease inhibitor	-0.02
Enzyme inhibitor	0.53

**Figure 4:**
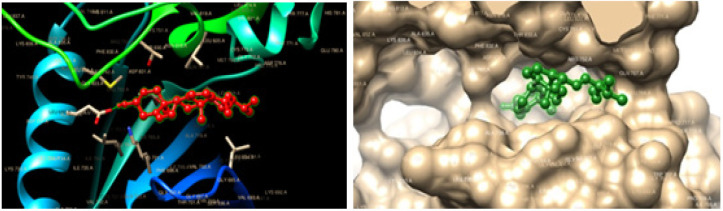
Docking Results of Stigmasterol in Epidermal Growth Factor Receptor (EGFRK) (PDB code: 1M17) (Pose view and Chimera 1.13.1 viewer)

**Figure 5:**
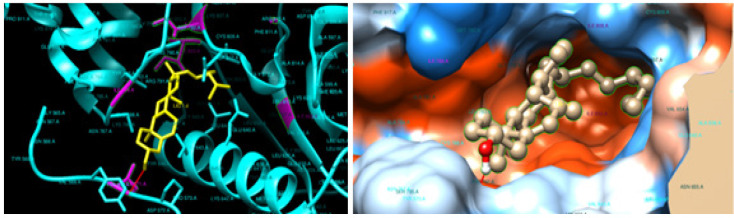
Docking Results of Stigmasterol in Protein Tyrosine Kinase (PTKs) (PDB code: 1t46). A-Method Validation using Crystallized and Docked Imatinib (Pose view and PyMol viewer) (Pose view and Chimera 1.13.1 viewer)

**Figure 6:**
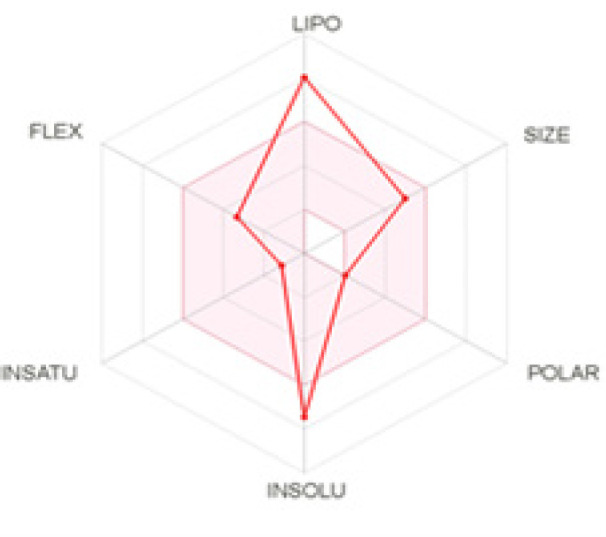
The Bioavailability Radar Enables a First Glance at the Drug-likeness of a Molecule
